# EQUIP Emergency: study protocol for an organizational intervention to promote equity in health care

**DOI:** 10.1186/s12913-019-4494-2

**Published:** 2019-10-10

**Authors:** Colleen Varcoe, Vicky Bungay, Annette J. Browne, Erin Wilson, C. Nadine Wathen, Kat Kolar, Nancy Perrin, Scott Comber, Amélie Blanchet Garneau, David Byres, Agnes Black, Elder Roberta Price

**Affiliations:** 1 Critical Research in Health and Healthcare Inequities Research Unit, School of Nursing, The University of British Columbia, Vancouver, BC Canada; 2 School of Nursing, University of Northern British Columbia, Prince George, BC Canada; 3 Faculty of Information & Media Studies & Arthur Labatt Family School of Nursing, Western University, London, ON Canada; 4 Johns Hopkins University School of Nursing, Baltimore, MD USA; 5 Rowe School of Business, Dalhousie University, Halifax, ON Canada; 6 Faculté des sciences infirmières, Université de Montréal, Montreal, QC Canada; 7 BC Ministry of Health, Victoria, BC Canada; 8 Professional Practice Office, Providence Health Care, Vancouver, BC Canada

**Keywords:** Health inequities, Health disparities, Stigma, Discrimination, Racism, Emergency, Intervention research

## Abstract

**Background:**

Social inequities are widening globally, contributing to growing health and health care inequities. Health inequities are unjust differences in health and well-being between and within groups of people caused by socially structured, and thus avoidable, marginalizing conditions such as poverty and systemic racism. In Canada, such conditions disproportionately affect Indigenous persons, racialized newcomers, those with mental health and substance use issues, and those experiencing interpersonal violence. Despite calls to enhance equity in health care to contribute to improving population health, few studies examine how to achieve equity at the point of care, and the impacts of doing so. Many people facing marginalizing conditions experience inadequate and inequitable treatment in emergency departments (EDs), which makes people less likely to access care, paradoxically resulting in reliance on EDs through delays to care and repeat visits, interfering with effective care delivery and increasing human and financial costs. EDs are key settings with potential for mitigating the impacts of structural conditions and barriers to care linked to health inequities.

**Methods:**

EQUIP is an organizational intervention to promote equity. Building on promising research in primary health care, we are adapting EQUIP to emergency departments, and testing its impact at three geographically and demographically diverse EDs in one Canadian province. A mixed methods multisite design will examine changes in key outcomes including: a) a longitudinal analysis of change over time based on structured assessments of patients and staff, b) an interrupted time series design of administrative data (i.e., staff sick leave, patients who leave without care being completed), c) a process evaluation to assess how the intervention was implemented and the contextual features of the environment and process that are influential for successful implementation, and d) a cost-benefit analysis.

**Discussion:**

This project will generate both process- and outcome-based evidence to improve the provision of equity-oriented health care in emergency departments, particularly targeting groups known to be at greatest risk for experiencing the negative impacts of health and health care inequities. The main deliverable is a health equity-enhancing framework, including implementable, measurable interventions, tested, refined and relevant to diverse EDs.

**Trial registration:**

Clinical Trials.gov #NCT03369678 (registration date November 18, 2017).

## Background

Social and health inequities are widening globally. Health inequities are unjust differences in health and well-being between and within groups of people caused by socially-structured, and thus avoidable, marginalizing conditions [1, 2]. In Canada, marginalizing conditions include an eroded social safety net, rising income inequality, deeply damaging impacts of historical and ongoing colonialism and racism, and enduring stigma and discrimination against people who experience issues such as disability, mental health or substance use [3]. Marginalizing conditions disproportionately position Indigenous persons, racialized newcomers, those experiencing poor mental health, substance use issues or interpersonal violence as vulnerable to a wide range of acute and chronic health problems, and create barriers to accessing care [4–11]. This results in inverse care, where those who experience social inequities and poorer health often have the least access to appropriate care, and are more likely to experience lower quality, under-resourced care. Inverse care may exacerbate harms, particularly for populations experiencing the negative impacts of structural inequities [10, 12–14]. As is the case globally, Indigenous people in Canada experience significant health inequities that are directly related to race-based colonial policies and racism, and exacerbated by discrimination across social institutions, including health care broadly and in emergency departments (EDs) specifically [15–20]. Research shows that discrimination towards Indigenous people in health care settings leads to misdiagnoses, under-treatment and medical errors, deters timely care and increases conflict, and increases costs and worsens outcomes [15, 17, 21–24].

Enhancing equity in health care is an important strategy to improve population health. While calls for equity in health care abound (e.g., [2, 25]), few studies examine how to achieve equity at the point of care, and the impact of doing so. Having identified the key dimensions of equity-oriented health care (EOHC) [7, 20, 26], we developed and tested an organizational-level intervention to promote equity at the point of care in primary health care (PHC) settings [6]. Study of this intervention, entitled “Equipping PHC for Equity (EQUIP PHC)” found that for patients, EOHC predicted greater comfort with and confidence in care, which in turn predicted greater confidence in managing their own health, and consequently better health outcomes, including fewer depressive and trauma symptoms, less disabling chronic pain and better quality of life [5]. We also showed that staff involved in EQUIP PHC had greater confidence and skills in providing EOHC [4]. Lessons from the primary care study indicated that supporting direct care providers to have greater ownership in implementing the intervention in their settings, delivering it more intensely over a shorter time frame, and preparing staff and leadership for inevitable disruptions in knowledge, attitudes and practices would strengthen intervention uptake and, ideally, impact [4]. We also learned that care providers wanted “tools” to help them translate the abstract ideas behind equity-oriented health care into action. Importantly, throughout our PHC study, numerous patients and most providers expressed concerns about patient’s negative experiences and difficulties accessing care in EDs. Negative experiences included stigma and discrimination based on racism, mental health issues, and substance use.

Emergency departments in Canada often operate at over-capacity [10, 11, 15, 27], in part because of the lack of primary health care services responsive to the needs of populations experiencing marginalization. While EDs are not designed to rectify shortfalls in primary health care, they nevertheless present a key arena for mitigating the impacts of structural conditions and barriers linked to health inequities at the point of care [5, 7, 15, 27]. However, despite intentions within health care systems to uphold principles of fairness, many people facing marginalizing conditions continue to experience inadequate and inequitable treatment in EDs, which makes people less likely to access care, paradoxically resulting in reliance on EDs through delays to care and repeat visits, interfering with effective care delivery and increasing human and financial costs [15–17, 21, 22, 28]. Improving care quality in EDs for people at greatest risk for health and social inequities is essential to reducing readmission rates, reducing admissions for ambulatory care-sensitive and family practice-sensitive conditions (e.g., COPD, respiratory infections, post-surgery care), improving continuity of care and cutting overall costs to the system [27]. Building on the lessons learned in our PHC study, we are adapting EQUIP to ED contexts and testing its impact at three geographically and demographically diverse EDs in one Canadian province. This paper outlines the study protocol for the EQUIP Emergency (EQUIP ED) Study and briefly describes the intervention framework.

### Approach and settings

EQUIP Emergency is a study of an organizational-level intervention to improve care quality at the point of care for those who face health inequities. This study is a three-way collaboration among health researchers, health care staff and Indigenous/community leaders aimed at developing an evidence-based intervention framework to promote equity for Indigenous and non-Indigenous people in diverse EDs. The study is part of a broader program of research entitled EQUIP Health Care that aims to reduce health inequities at the point of care in pursuit of the quadruple aims of health system optimization: improving the health of populations, enhancing patient experiences and outcomes, reducing per capita cost of care, and improving the work life of staff [29, 30]. A key goal is to create intervention processes that are, if evaluated to be effective, scalable and “ready to implement.” As such, our approach to knowledge mobilization, broadly defined as “a wide range of activities relating to the production and use of research results, including knowledge synthesis, dissemination, transfer, exchange, and co-creation or co-production by researchers and knowledge users,” [31] is both integrated and grounded in key principles of implementation science [32, 33].

Approaching EDs as complex adaptive systems [34], this mixed-methods multisite study will:

1. engage ED staff and leaders in participatory implementation processes to enhance organizational capacity for EOHC;
2. examine impacts of EQUIP on processes of care, patient experiences of care and short-term outcomes, organizational policies, and on staff attitudes, confidence, behaviours and job satisfaction;
3. analyze the cost-benefit and feasibility of EQUIP activities and scale-up potential in other contexts.

A tripartite model of Indigenous/community, practice and research team members is used throughout. The research leadership team is comprised of an Indigenous Elder with extensive research experience (author RP), a health care leader with wide-ranging expertise in practice leadership and research (author DB), and four academic researchers (authors CV, VB, AJB, EW) as co-Principal Investigators. Additional co-investigators are diverse Indigenous, practice and research leaders. The project is guided by an advisory panel, comprised of Indigenous community, practice and research leaders. For all intervention activities, the research team is supporting the engagement of Indigenous and community leaders, health care leaders and all staff in each ED.

We are working with 3 EDs in one Canadian province, purposely selected to capitalize on our extant research relationships and maximize diversity of settings: an inner-city, a suburban area, and a northern region serving rural, remote and small urban communities. We are developing, implementing, testing and refining the framework to optimize care for Indigenous people and a wider range of people who commonly face stigma and discrimination. We have designed an overall process that will be tailored to the unique characteristics, context and strengths of each ED. For example, one ED has extensive expertise providing care to patients with substance use problems, another has expertise caring for diverse groups of new immigrants, and one is located in a region with relatively high proportions of Indigenous populations. Collaborating with organizational leadership in these diverse health authorities promotes understanding of the potential scale up to other EDs. Table 1 provides some details regarding each.

**Table 1:** Overview of Study Sites: Key equity-relevant features

Study Site	Health Authority	Key Equity-Related Characteristics
St. Paul’s Hospital Vancouver, British Columbia (BC)	Providence Health; affiliated with Vancouver Coastal Health	• Located on un-ceded traditional lands of the xʷməθkʷəy̓əm (Musqueam), Skwxwú7mesh (Squamish), and Səl̓ílwətaʔ/Selilwitulh (Tsleil-Waututh) Nations • Primary hospital serving people living in the inner-city neighborhood known as the Downtown Eastside (DTES) of Vancouver • DTES residents experience some of the highest levels of health and social inequities in Canada (e.g., no fixed address, malnutrition, complex medical problems such as HIV) • Part of a Catholic health care community with a strong history of social justice • Located in the epicentre of the opioid overdose crisis in Canada; high proportions of patients experience significant substance use issues • Percentage of people identifying as Indigenous varies, with fewer than 2% in the gentrified areas, and up to 30% in others [35]
Surrey Memorial Hospital Surrey, British Columbia	Fraser Health	• Located on the lands of the Semiahmoo, Katzie, Kwantlen, Tsawwassen, QayQayt and Kwikwetlem First Nations • The largest ED in Western Canada, with approximately 140,000 patient visits per year • Serves the highest concentration of newcomers in BC (43%), including people who immigrated from India (41%), China (15%) and the Philippines (13%) [36]
University Hospital of Northern British Columbia Prince George, British Columbia	Northern Health	• Located on the traditional territory of the Lheidli T’enneh First Nation • Level III trauma centre, providing services to people dispersed over an area of 600,000 km^2^ in northern BC • Advanced referral ED for over 300,000 residents of diverse rural, remote and isolated communities • 20.1% of population served identifies as Indigenous (Indigenous people comprise 4.9% of the Canadian population overall) [36]

### Theoretical approaches and evidence base

Our understanding of equity and EOHC is informed by critical theoretical understandings of social justice, and the structures that perpetuate health and social inequities. The overall study is guided by complexity theory using an integrated approach to implementing and mobilizing interventions. These approaches and our tripartite leadership model are complemented by a change leadership approach known as Front Line Ownership (FLO), all of which align with understanding health care systems and EDs as complex adaptive systems.

### Complexity theory

Intervention research to bring about change within health care settings has historically had low success rates. Lack of success has been associated with failing to attend to the complexity of environments in which interventions are carried out, or factors associated with capacity and readiness for change [37–39]. Consequently, the intervention detailed in this protocol was influenced by central tenets of complexity theory. As noted, EDs are complex adaptive systems with many component parts that interact and influence one another [39]. These interactions include and extend beyond the patient level to the provider, organization, and policy levels of health care [40]. Complexity theory directs us to analyze the diverse and complex interactions among these components.

### Front line ownership

In addition to attending to the complexity of the ED settings, our intervention design utilizes the evidence-based and theoretically-informed change management approach of Front Line Ownership. With its roots in complexity theory, FLO is an intentional departure from prevalent hierarchical health care change management practices whereby leaders promote ideas to staff and try to get buy-in. Instead, FLO involves those most actively engaged in direct patient care developing the ideas, making the decisions and designing and acting on the plans they create [41, 42]. FLO includes change processes that support participatory, peer-based learning and recognizes that learning and change are most effective and sustainable when the solutions (in this case, to the problem of inequities in health and health care) are generated by the health care staff themselves [42]. FLO does not infer absence of formal change leadership; it requires that leaders support the development of staff capacity to both identify and lead change initiatives. Supportive strategies, often referred to as “liberating structures,” [43] include quick-to-learn tools for the staff to enhance their capacity for facilitation among their peers including active listening, engagement and inclusion of ideas and strategies among the diversity of staff working in the setting (e.g., nurses, physicians, security personnel, housekeeping, unit coordinators or admitting clerks). FLO supports staff to both identify and think about problems differently and to take a leadership role to generate solutions to problems that occur in their settings [43]. FLO achieves increased change sustainability by: identifying local solutions that reflect the unique context in which issues arise; increasing organizational interconnectivity and the development of new action pathways via a process of point of care, worker-directed collaboration; and building the capacity of these workers for real-time problem-solving over time [42]. FLO is a contextual and non-linear approach to change management and change leadership ideally suited to interventions occurring in complex settings.

### Equity-oriented health care

At the definitional core of health equity is the critical analysis of power and the workings of discrimination dynamics in the pursuit of social justice [4, 44, 45]. Our conceptualization of EOHC is grounded in understanding discrimination as a socially structured and often implicitly sanctioned phenomenon, justified by ideology and expressed in interactions among and between individuals and institutions in ways that maintain privileges for members of dominant groups, and contribute to inequities for others [13, 46], with profound impacts [47]. Experiences of discrimination are often amplified when issues of poverty, substance use, or stigmatizing conditions such as HIV or chronic pain syndromes intersect with people's sense of being treated differentially on the basis of their ethno-cultural and other identities [48]. From this vantage point, interventions aimed at improving organizational cultural competencies or providing diversity education for service providers to reduce discrimination and increase access to services are often good starting points, but are insufficient to achieving equity-oriented practices. For example, such interventions often lack a focus on how social systems produce and sustain health inequities, thus allowing the fundamental causes of inequities to remain unidentified and unchallenged. In comparison, equity-oriented approaches seek to address the continuities between structural and interpersonal discrimination. Such approaches go beyond providing education to fill knowledge gaps, and aim to support critical reflection and action by providers and other staff on the interconnected barriers to health care across personal (e.g., stereotypes and assumptions about patients), interpersonal (e.g., interactions with patients that may be discriminatory), organizational (e.g., security protocols that create access barriers for patients who experience poverty, mental health or substance use issues) and structural levels (e.g., inadequate access to housing and social welfare supports).

EQUIP PHC, the precursor to the current study, showed that in PHC settings EOHC was associated with positive health outcomes [5], and that intervening at the organizational level improved staff confidence and comfort with providing such care [4]. We do not, however, know whether such an intervention can *improve* health outcomes, or how emergency department staff can be supported to provide EOHC. This project pursues three key dimensions for enacting equity-oriented health care derived from our prior research [6, 7, 20]: Culturally-Safe Care; Trauma- and Violence-Informed Care (TVIC); and Harm Reduction, each of which must be Contextually Tailored. These key dimensions, described in greater detail in previous publications [4], are briefly summarized below.

*Culturally-Safe Care* locates the culture of health care as the site for transformation and seeks to challenge discriminatory values and assumptions held within health care across interpersonal, historical, and structural levels (e.g., legacy of colonization and racism that shapes discrimination as a barrier to health care) [4, 7, 20]. It shifts the focus away from attempting to identify the unique cultural characteristics of individuals or groups as the potential barriers to care, towards critiquing structural conditions and inequitable power relations, requiring changes to dominant norms to enhance care for everyone.

*Trauma- and Violence- Informed Care (TVIC)* involves understanding past and ongoing interpersonal and structural violence as causes of trauma, and developing policies and practices to minimize harm, prevent re-traumatizing people during care, and promote healing. The primary goal of TVIC is to create safe and trusting environments [49].

*Harm Reduction* involves practices that mitigate harms, not only of substance use but also harms associated with the historical, socio-cultural and political determinants of substance use and related social and health policy [50].

*Contextually-Tailored Care* explicitly and continually adapts services, practices, organizational policies and clinical guidelines to optimally address the populations served and to local social and community contexts, thus expanding beyond the individually-focused concept of patient-centred care [4].

## Methods/design

Based on the above theoretical and empirical innovations, EQUIP Emergency is an organizational intervention tailored to hospital emergency department settings. Drawing on the concept of FLO, all categories of staff involved in providing patient care in each ED (e.g., security, nurses, clerks, cleaning staff, physicians, aides) will be invited to participate in self-led working groups over a 6-month catalyst period. Working groups will assume a leadership role in facilitating change within their locales, identifying and prioritizing areas of improvement to foster equity among patient populations depending on the context. With the goal of recruiting direct-care staff to participate in working groups, we will begin with information sessions, flyers, emails and meetings to provide orientation to the project. In line with contextual tailoring, these communication methods will build on preferred site-specific practices. For example, one site typically holds 10-min staff huddles to share information, and these will be used to share information and recruit working group members at that site. Administrative leadership at all sites has committed to paid staff time for meetings and intervention-related activities. Working groups will be provided with:

- an orientation to EOHC, envisioned as a 2–3 h workshop;
- access to the online *Equipping Health Care for Equity Modules* and tools (https://equiphealthcare.ca/modules);
- a context profile of the communities served including demographics and history, with an emphasis on those most likely to experience social and economic marginalization;
- data snap shots from patient and staff survey data collected as part of the research;
- access to two types of coaches to support participants to apply the three key dimensions of EOHC and tailor such application to their contexts:
  - A *change coach* at each site will encourage participants to identify social, political, and economic forces that restrict possibilities for change, to reflect on the interpersonal and emotional impacts of organizational change, and to develop creative solutions in light of restrictions and potential disruptions. Having learned in our PHC research that efforts to promote EOHC were necessarily disruptive, change coaches will help teams to anticipate such disruption toward having the most positive impact possible [4].
  - *Content coaches* will encourage participants to reflect on how systemic inequities impact the health and wellbeing of people at the population level, the barriers to health care that people experience, and interpersonal interactions. They will also support the working groups to think creatively about strategies that might mitigate such inequities. Given the diverse expertise required, there may be several content coaches; for example, one with expertise in harm reduction, another in Indigenous-specific cultural safety.

Ultimately, the coaches will work to render themselves unnecessary to the working groups.

- a work book to guide the working group activities overall. The work book is based on principles derived from the theory and evidence underpinning EQUIP, and will lead working groups through 5 steps: 1) orienting themselves to EOHC, 2) planning how to work together, 3) assessing their department, 4) planning change initiatives, and 5) evaluating and monitoring. Assessment of the department is organized around a SWOT (Strengths, Weaknesses, Opportunities, and Threats) heuristic. To facilitate the SWOT analysis, working groups will be able to draw summaries of patient and staff data from our baseline data collection, and the context profile. Patient demographics, compared to population demographics, and patients’ experiences of discrimination and ratings of care will be analyzed according to pertinent subgroups (e.g., those identifying as Indigenous or those with unstable housing) to help staff identify groups who may be best and least well served. Having learned in our PHC study that staff found explicit analysis of the impact of their population demographics invaluable [51], we see such analysis as foundational to equity-promoting change.

The change process will be facilitated with 1) paid staff time for working group members to attend meetings for 6 months (anticipated to be monthly or semi-monthly two-hour meetings), 2) a site-specific research assistant (2 hours/week), 3) access to change and content coaches for up to 20 h total over 6 months and 4) a $10,000 catalyst grant to be used for change initiatives. The employers have committed to paying staff time, and the remaining resources are provided by the research funding. The working group members will decide the focus and duration of coaching with a minimum of an introductory session with a change coach.

### Research design

**Fig. 1:**
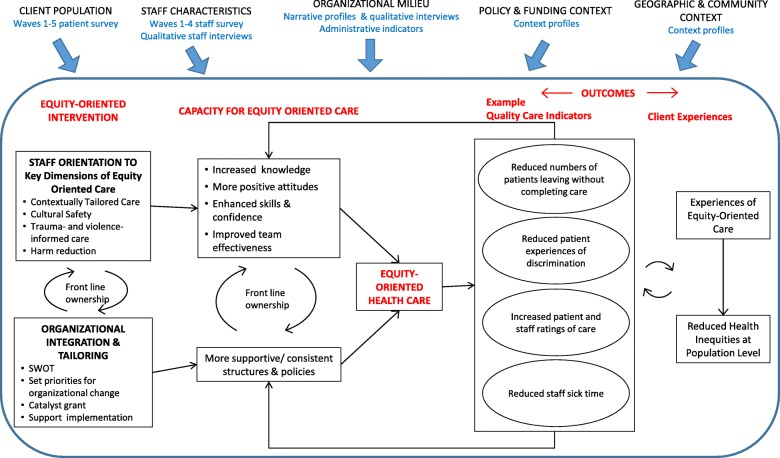
Intervention Theory

A mixed methods multisite design will examine changes in key outcomes specified in the intervention theory (Fig. 1) including: a) a longitudinal analysis of change over time based on structured assessments of patients and staff, b) an interrupted time series design (ITS) of administrative data (i.e., staff sick leave, patients who leave without care being completed, including those who register but leave before being seen by a care provider, before being assessed, before being treated, or against care provider advice), c) a process evaluation to assess how the intervention was implemented and the contextual features of the environment and the process that are influential for successful implementation, and d) a cost-benefit analysis. Table 2 provides a summary of our hypotheses and research questions and the related data sources and analytic approaches.

**Table 2:** Research questions & data sources

Hypothesis/Question	Data Sources	Analysis
Patient overall ratings of care will improve from pre-intervention to post-intervention and be sustained at 6 & 12 months Patient self-reported experiences of discrimination will decrease from pre-intervention to post-intervention and be sustained at 6 & 12 months	Patient Survey	Generalized Estimating Equations to test change over time
Staff perceptions of care and team effectiveness will improve pre-intervention to post-intervention and be sustained at 6 & 12 months	Staff Survey	Generalized Estimating Equations to test change over time
The number of patients leaving without care being completed will decrease pre-intervention to post-intervention and be sustained at 6 & 12 months	Administrative Data	Interrupted Time Series with segmented regression
Staff sick time will decrease pre-intervention to post-intervention and be sustained at 6 & 12 months	Administrative Data	Interrupted Time Series with segmented regression
What is the impact of EQUIP ED on organizational policies and practices?	Observations Qualitative interviews Policies	Ethnographic analysis
What influences the uptake of EQUIP ED?	Observations Qualitative interviews Documents Policies	Process Evaluation

### Longitudinal analysis of change

Changes in patients’ overall ratings of care (primary outcome), and self-reported experiences of discrimination in the emergency department (secondary outcome) will be examined using survey data collected at 5 points in time: 2 times pre-intervention, immediately post-intervention, and at 6 months and 12 months post-intervention. Staff perceptions of patient care (primary outcome) and staff satisfaction and engagement with their job and workplace (secondary outcome) will be examined using survey data collected at 4 points in time: pre-intervention, immediately post-intervention and 6 and 12 months later. Table 3 provides a summary of the patient and staff survey items and their sources.

**Table 3:** Overview of Patient and Staff Surveys

	Measure	Number of items	Source & References
Patient survey data	Discrimination in Everyday Life	8	Everyday Discrimination Scale [52]
	Discrimination during ED Visit	7	Discrimination in Medical Settings Scale [53]
	Experiences of Care	32	ED Patient Experience of Care survey [54] and BC EDPEC [55] 7 items developed for EQUIP ED Study
	Demographics	4	Rainbow Health Ontario’s Sexual Orientation Measures [56] Canadian Community Health Survey [57] Financial Strain Index [58]
Staff survey data	Your Work Experiences	25	Accreditation Canada’s Worklife Pulse Tool [59] 1 item developed for EQUIP ED Study
	Team Effectiveness	11	Canadian Institute for Health Information’s PHC Team Effectiveness Scale [60]
	Perceptions of patient care	1	Developed for EQUIP ED Study
	Cultural Safety	5	All items developed for EQUIP Research
	Trauma- and Violence- Informed Care	5	All items developed for EQUIP Research
	Care related to Drug and Alcohol use	7	All items developed for EQUIP ED Study

### Interrupted time series (ITS)

Data will be collected at equally spaced intervals (monthly) on selected variables hypothesized to be sensitive to EOHC, and which have sufficient stability over time. Variables include the number of people who leave the ED prior to the completion of care (primary outcome) as a percentage of the total number of people seen, and the rate of staff sick time taken as a proportion of productive hours (primary outcome). Using retrospective data, we will collect monthly data from hospital administrative sources for 24 months prior to the start of the intervention, 12 months during the implementation of the intervention and 12 months post-intervention for each variable.

### Process evaluation

From a complex systems perspective, a research intervention is seen as an event that unfolds within and occurs in interaction with a system [38, 39, 61]. In order to accurately document and assess the implementation of an intervention and the relevance of the implementation processes for intervention outcomes, it is necessary to monitor the contextual influences shaping intervention activities. A process evaluation will be conducted to examine how contextual influences shape EQUIP Emergency implementation and ultimately, outcomes, including the readiness of the EDs to engage in activities to foster EOHC. Table 4 provides a summary of the proposed process evaluation phases mapped across EQUIP intervention activities and key data sources.

**Table 4:** Process Evaluation Matrix

	Enablers	Component/Activity	How or why are we doing this?	Key Performance Indicators	Data Sources
Pre-Intervention *Assessing Readiness and Engagement*	Change Readiness, Leadership & Policy, Engagement	Engage hospital leadership in project	Secure leadership buy-in & support for grant; Assess change readiness of site; Build leaders’ knowledge of core principles (EOHC, FLO)	• Commitment from hospital leadership to participate in project • Agreement from leadership to sign on to grant application	• Qualitative interviews • Meeting minutes
	Leadership & Policy, Funding, Engagement	Secure fiscal commitment from sites	Fund working group staff time; Support FLO in intervention	• Stated commitment to fund Working group at each site	• Qualitative interviews • Meeting minutes
	Leadership & Policy, Engagement	Build rapport with unit leaders	Assess change readiness of unit; Foster participation & support for project	• Stated commitment to participate in project	• Qualitative interviews • Meeting minutes
Intervention PHASE I *Working Group Engagement*	Engagement Knowledge & Training	Spread awareness of EQUIP project at unit level	Foster awareness of EQUIP research activities; Begin to generate interest in working group; Lay groundwork for working group activities	• Diverse categories of staff attend orientation sessions • Communications received by research team about the study by ED staff	• Field notes • Qualitative interviews
	Change Readiness, Engagement Enrollment, Front Line Ownership, Stewardship	Facilitate staff interest in working group	Facilitate development of working group; Foster FLO processes; Prepare for intervention activities	• Working group forms at each site	• Field notes • Qualitative interviews
Intervention PHASE II A *Establishing a Sustainable Working Group*	Knowledge & Training, Front Line Ownership, Stewardship	Identification of change coaches and equity coaches to support working group activities	Support the working group’s change process: translating assessments to action, anticipating and managing disruption	• Identification of equity coach and change coach for each site • Finalized job description for change coaches	• Qualitative interviews
	Funding, Engagement, Human Resources Enrollment, Knowledge & Training, Data & Information, Front Line Ownership, Stewardship	Deliver orientation workshop during first working group meeting	Orient working group to core principles of EOHC & FLO; Foster FLO & project ownership; Facilitate working group team building; Provide roadmap for project activities (e.g. assessment)	• Staff knowledge of core principles • Staff commitment to core principles • Staff commitment to continue working group	• Field notes • Meeting minutes • Qualitative interviews
	Funding, Engagement Enrollment, Front Line Ownership, Data & Information Stewardship	Working group meets a second time to prepare for action	Foster FLO & project ownership; Provide opportunity to reflect on first meeting; Facilitate working group preparing for action	• Working group sets future meeting date(s) • Working group develops plan for further ED assessment	• Field notes • Meeting minutes • Qualitative interviews (working group members, coaches)
Intervention PHASE II B *Working Group Begins Action*	Funding, Engagement, Human Resources Knowledge & Training, Front Line Ownership, Stewardship	Working group develops internal mechanisms for supporting team (terms of reference, mandate)	Fostering FLO throughout intervention; Supporting success of working group intervention activities by building a functioning team	• Working group develops documents to guide teamwork throughout intervention	• Field notes • Meeting minutes • Qualitative interviews
	Change Readiness, Funding, Human Resources Knowledge & Training, Front Line Ownership, Stewardship	Working group identifies problem(s) and begins action to address this problem	Working group begins locally tailored intervention activities grounded in FLO principles	• Working group conducts equity walk-through(s) • Working group completes ED assessments, through existing data analysis and further assessment • Working group identifies key challenges to equity within each site	• Field notes • Meeting minutes • Qualitative interviews
	Engagement, Human Resources Front Line Ownership, Stewardship	Research team phases out leadership	Ensure FLO is operationalized throughout intervention activities	• Working group organizing and facilitating their own meetings • Research team in note-taking role • Working group members directing research team involvement • Working group identifies own knowledge and resources needs	• Field notes • Meeting minutes • Qualitative interviews
	Data & Information	Research team decides when to collect first post-intervention data	Ensure data collection is timely and according to study design, while also best capturing outcomes from progress toward stated goals at each site	• Dates finalized for post-intervention data collection at each site	• Meeting minutes
Intervention PHASE III *Working Group Implementation*	Funding, Leadership & Policy, Human Resources Knowledge & Training, Data & Information, Front Line Ownership, Stewardship	Working group develops project plan and budget for hospital/unit leaders and research team	Support FLO principles throughout intervention while ensuring accountability of working group for catalyst grant and project activities	• Working group submits project plan and budget to hospital/unit leaders and research team • Research team confirms release of funds to working group • Hospital/unit leaders provide feedback on project plan	• Field notes • Meeting minutes • Qualitative interviews • Project plan & budget documents
	Change Readiness, Funding, Human Resources Knowledge & Training, Front Line Ownership	Working group spends $10,000 catalyst grant to support activities		• Working group spends catalyst grant funds	• Field notes • Meeting minutes • Expense reimbursements • Working group 6-month progress report
	Change Readiness, Leadership & Policy, Funding, Engagement, Human Resources Knowledge & Training, Front Line Ownership	Working group implements identified equity strategies	Drive the intervention, supported by core principles of EOHC & FLO	• Working group communicates equity strategy plan to hospital/unit leaders • Working group communicates equity strategy plan to staff • Working group spends catalyst grant funds toward equity strategies	• Field notes • Meeting minutes • Qualitative interviews • Working group 6-month progress report
	Engagement, Human Resources Knowledge & Training, Front Line Ownership	Working group conducts peer evaluation to contribute to assessment of team effectiveness	Provide feedback to working group regarding team effectiveness to date; Support adaptation of working group to emergent needs and contribute to success of intervention activities; Contribute to success of working group team beyond 6-month intervention period	• Working group completes peer evaluation • Working group adapts team mechanisms and processes based on feedback from peer evaluation	• Field notes • Meeting minutes • Peer evaluation documents (researcher use of documents as data to be negotiated with working group) • Qualitative interviews

### Cost-benefit analysis

The costs of the intervention will be calculated from budget monitoring data for the research budget and for the costs of staff time. The cost-benefit ratio will be computed for each of the primary outcomes. These cost-benefit ratios will reflect the cost to improve patient and staff ratings of care by 1 unit, cost to have one less patient leave without care being completed, and cost to reduce each hour of staff sick time.

### Power analysis

The power analysis is based on the within-site analyses. For the longitudinal patient survey data, we estimated the number of patients needed per site to detect a small to moderate effect size of 0.25. Means and standard deviations from the EQUIP PHC study for patients’ experiences with care informed the power analysis. With power of 0.80 and alpha of 0.05, we can detect significant changes with an effect size of 0.25 with a sample size of 250 patients. We used methods suggested by Zhang et al. [62], to estimate the power for the ITS design. Based on the trend over time in the primary ITS outcomes extracted from the administrative data for the past 24 months, we estimate that the time series will have an autocorrelation of − 0.20 and will require an autoregressive model with 1 lag (AR1). Power is 0.85 to detect a moderate effect size with alpha of 0.05, AR1 model, and an autocorrelation of − 0.20 with 24 time points prior and 12 time points post the intervention.

### Data collection

#### Patient survey data

At each time point, at least 250 patients entering the ED will be recruited and consented. To simultaneously mitigate the risk of staff deviating from usual practice on data collection days and to recruit diverse patients including those without phones, over approximately 2 weeks (depending on patient flow), all adult patients who are able to give consent will be invited to participate as they present for care. Triage staff will hand patients an invitational flyer with study information. If patients are unable to give consent when first presenting for care (e.g., unconscious, in pain) but become able subsequently, they will be approached to participate when it appears they may be able to consent. Contact information will be collected from interested patients, who either will be interviewed immediately following discharge in a private interview room close to the ED, in their hospital room if admitted, or interviewed by phone within 5 days of their visit. Following processes we previously have used, we will collect data directly on tablets. We will select recruitment periods across all shifts and days of the week. Anticipating a 50% refusal rate [63] and 15–30 min per interview we will select the number of shifts required at each site.

#### Staff survey and interview data

We will conduct quantitative surveys focused on staff EOHC confidence, work experiences, and team effectiveness. Surveys will be open to all staff at four time points (baseline, at the end intervention and 6 and 12 months later). The number of staff varies greatly with each site (from ~ 80 to ~ 500), and we will aim for a survey response rate of 50%. In addition, we will conduct qualitative interviews with a range of staff pre- and post-intervention at each site, including staff who take leadership roles and those who express interest, but do not participate.

#### Leadership interview data

Semi-structured interviews will be conducted with research team leads, and with health authority leadership to assess the broader contextual influences affecting the acceptability and implementation of the intervention.

#### Observational data

Observations of the day-to-day operation of the emergency department and of the intervention activities at each site, including working group meetings and change initiatives, will be collected as field notes by those recruiting for and/or collecting patient and staff survey data, and site-specific research coordinators.

#### Administrative data

Data will be extracted from existing databases held by the EDs’ respective health authorities as well as the private firm contracted to provide security services to the hospitals. In addition to the primary outcome variables (patients who leave without care being completed, and staff sick time as a proportion of productive hours), variables of interest include equity-relevant incident reports (e.g., patient-initiated complaints), equity-relevant adverse event reports (e.g., violence, readmission rates, security incidents including verbal and physical aggression, threat assessments, and code whites), and staff indicators (e.g., staff turnover). These data will be extracted monthly for 24 months prior to the intervention, 12 months during implementation, and 12 months post-intervention implementation.

#### Documents

Documents produced by the research team, such as presentations and reports to the working groups, and documents produced by the working groups, such as meeting minutes and catalyst grant proposals will be collected. Unit and organizational policies and reports will be collected as they become relevant; for example, if a particular policy is identified as a barrier to equity and considered for revision.

#### Costs

Costs of the intervention will be calculated using data from research budgets and estimates of paid staff time. Intervention costs will include time and material costs associated with engaging the staff, staff time for the working groups and any paid staff time for intervention activities, coaches’ time, work books, cost of time for the research team to generate reports for each ED, and the catalyst grants.

### Data analysis

Longitudinal analysis will be conducted within site and for all three sites combined. For each wave of patient and staff survey data, descriptive statistics appropriate to the level of measurement will be computed. Analysis of change across time will use General Estimating Equations (GEE) to account for the repeated measures over time. Time (5 levels for patient survey data and 4 levels for staff survey data) will be the independent variable. We hypothesize increases in patients’ mean ratings of care and in staff perceptions of care (primary outcomes), and decreases in patient experiences of discrimination in the ED and increases in staff engagement and team effectiveness (secondary outcomes).

For the analysis of the ITS data, segmented regression with autoregressive models will be used [64]. Interrupted time series are tested by comparing the level of the outcome of interest and the rate of change over time between the time period prior to the system change and the time period after the system change [65]. The model will yield an estimate of the level and slope across time of the outcomes prior to the intervention and changes in the level and slope after the intervention. The change in level provides an estimate of the immediate effect of the intervention, and the change in slope provides an estimate of the ongoing effect of the intervention across time after implementation. We hypothesize that there will be a significant change immediately after the intervention with a continued improving trend.

A cost per unit of improvement will be calculated for each of the primary outcomes. Improvement will be the change in the outcome from the time point immediately prior to the start of the intervention implementation to the 12 months after the intervention. Total cost will be divided by the change in outcome and scaled to reflect the cost per 1 unit improvement in patient and staff perceptions of care. For the number of patients who leave without care being completed, the difference in the total number of patients who left in the 12 months prior to and post-intervention will be computed. Total cost to deliver the intervention will be divided by this number to reflect the cost to achieve one fewer patient per year leaving without care being completed. This same approach will be used with respect to staff sick time (total staff sick time in the 12 months prior compared to the 12 months post-intervention).

Process evaluation analysis will be conducted for each site and then combined to assess the contextual features influencing intervention implementation at organizational (e.g., leadership within the health authority and specific health care facilities) and unit (e.g., emergency department staff and structures) levels. Each phase detailed in Table 4 will be analyzed first at each site to identify the degree to which the activities have achieved the intended purpose using the predetermined performance indicators for enablers assumed to foster successful implementation. Thematic analysis will be conducted using data from meeting minutes, field notes, and qualitative interviews to explain the outcomes of this analysis. Results from the analyses at each site will be combined and analyzed to identify similarities and differences across sites including potential rationale for any discrepancies. The analysis will be refined, advancing theoretical coding to assess the barriers and enablers related to the core theoretical tenets underpinning the intervention including front-line ownership as a change management approach and equity-oriented health care and its related dimensions. This analysis will identify the processes that facilitated or hindered the implementation of EQUIP Emergency and the circumstances under which EQUIP is likely to be successful, thereby providing important information about scale-up potential.

### Ethical issues

Key ethical issues are the prioritizing of care processes over research processes, the protection of privacy and ensuring informed consent for patients and staff, and ensuring that research processes do not compromise staff employment relationships. The emergency departments serving as study sites are very busy and often operate at over-capacity. In this context, patient care will be the top priority and no research activities will interfere with care. Efforts to ensure privacy will include a) using tablets for patient surveys and online surveys for staff, b) supporting patients to enter their own data directly, or at least read the questions (without them being read out loud so that others can overhear), c) using private spaces to complete surveys when this is not possible, and conducting all qualitative interviews with staff and leaders in private spaces. The EDs serve diverse populations, thus efforts to ensure informed consent for patients will include a) reading aloud study information and consent letters unless the patient indicates this is not needed (not assuming literacy), b) offering materials translated into the top two languages at each site (for a total of 4 languages: English, Hindi, Punjabi and Cantonese). All study processes have been reviewed and approved by the Research Ethics Boards at the University of British Columbia and each Health Authority.

## Discussion

This project will generate both process- and outcome-based evidence to improve the provision of equity-oriented health care in emergency departments, particularly targeting groups known to be at greatest risk for experiencing the negative impacts of health and health care inequities. The main deliverable is EQUIP’s health equity-enhancing framework, including implementable, measurable interventions, tested, refined and relevant to diverse EDs. Importantly, the EQUIP framework integrates innovative, evidence-based strategies to mitigate discrimination and racism experienced by Indigenous people, and potentially faced by people of all ethnicities related to substance use, housing instability, sex work, gender, or mental illnesses. In addition to improving care delivery, this project contributes new knowledge about: how to improve health outcomes for vulnerable groups (e.g., improved fit of care with need, fewer people leaving without care being completed); and how to implement new interventions and mobilize knowledge arising from testing complex health equity-promoting interventions. Analyzing the effectiveness of implementing EQUIP in EDs and the associated cost-benefit will generate knowledge about the potential for scale-up of EQUIP in settings beyond the ED.

## Data Availability

Not applicable

## Abbreviations

DTES: Downtown Eastside
ED: Emergency Department
EOHC: Equity-Oriented Health Care
EQUIP ED: EQUIP Emergency Study
EQUIP PHC: Equipping Primary Health Care for Equity Study
FH: Fraser Health
FLO: Front-Line Ownership
GEE: Generalized Estimating Equations
ITS: Interrupted Time Series
PHC: Primary Health Care
SWOT: Strengths, Weaknesses, Opportunities, and Threats
TVIC: Trauma- and Violence-Informed Care
